# International Trauma Questionnaire and Posttraumatic Cognitions Inventory-9: validity evidence and measurement invariance of their Brazilian versions

**DOI:** 10.1186/s41155-024-00297-z

**Published:** 2024-05-07

**Authors:** Isabelle Aprigio, Pedro Paulo Pires dos Santos, Gustavo Gauer

**Affiliations:** 1 Federal University of Rio Grande do Sul (UFRGS), Rua Ramiro Barcelos, 2600, Room 227, Porto Alegre, RS CEP 91900-410 Brazil; 2 Federal University of Rio de Janeiro (UFRJ), Rio de Janeiro, Brazil

**Keywords:** Psychological trauma, ITQ, PTCI, Validity evidence, Invariance

## Abstract

**Background:**

The International Trauma Questionnaire (ITQ) is used to measure posttraumatic stress disorder (PTSD) and complex posttraumatic stress disorder (CPTSD) symptoms, and the Posttraumatic Cognitions Inventory-9 (PTCI-9) is used to measure posttraumatic cognitions. Both tools have been translated for use in Brazil. However, the psychometric properties of the Brazilian versions were not investigated, and no study has verified the invariance of these tools for many traumatic event types.

**Objective:**

This study examined the validity, reliability, and measurement invariance of the Brazilian versions of the ITQ and the PTCI-9 for trauma type, gender, race, age group, education level, and geographical region.

**Methods:**

A total of 2,111 people (67.74% women) participated in an online survey. The scale models were tested via confirmatory factor analyses and measurement invariance through multigroup analyses. Pearson’s correlation analyses were used to examine the relationships between PTSD, CPTSD, posttraumatic cognitions, and depressive symptoms.

**Results:**

Except for the affective dysregulation factor, the reliabilities of the ITQ and PTCI-9 dimensions were adequate. Models with six correlated dimensions for the ITQ and three correlated dimensions for the PTCI-9 showed adequate fit to the data. The ITQ and PTCI-9 exhibited scalar invariance for gender, race, age group, education level, and geographical region. The ITQ also demonstrated full invariance for trauma type. The factors of both instruments were related to each other and to depressive symptoms, with higher effect sizes for posttraumatic cognitions and complex posttraumatic stress disorder symptoms.

**Conclusion:**

We recommend using the Brazilian versions of the ITQ and PTCI-9, which are crucial tools for assessing and treating trauma-related disorders.

**Supplementary Information:**

The online version contains supplementary material available at 10.1186/s41155-024-00297-z.

Most people will experience life events that are highly stressful or traumatic. A recent review of prevalence studies (Schein et al., 2021) revealed that 8–84% of people exposed to such events will develop posttraumatic stress disorder (PTSD). PTSD encompasses symptoms of intrusive reexperiencing of a traumatic event, accompanied by intense emotions, avoidance of event-related stimuli, and a state of generalized hypervigilance (WHO, 2018). Moreover, individuals who experience traumatic events present distorted beliefs about themselves (e.g., “I have no future”), the world (e.g., “People can’t be trusted”), and their levels of blame for the occurrence (e.g., “The event happened because of the way I acted”; Foa et al., 1999). In terms of associated clinical conditions, depression is among the most prevalent comorbidities in individuals with PTSD and posttraumatic cognitions (Camden et al., 2023; Serier et al., 2023).

There is a consistent relationship between trauma-related cognitions and PTSD severity (Gómez de La Cuesta et al., 2019). For the measurement of trauma-related cognitions, Foa et al. (1999) developed the Posttraumatic Cognitions Inventory (PTCI) based on emotional processing theory. The theory focuses on the idea that traumatic life events modify a person’s beliefs, influencing later emotional responses. Effective treatments for PTSD impact the reduction of posttraumatic cognitions, and, commonly, this reduction precedes symptom remission (Brown et al., 2019), thereby corroborating the relevance and potential mechanism of changes in beliefs about the self, about the world and self-blame in PTSD.

Posttraumatic cognitions have a more substantial impact on traumatic events that involve interpersonal aggression, with minimal or no chance to escape, and that last long periods (e.g., child abuse, bullying, sexual abuse). Victims of those types of trauma report having more negative cognitions about the self, the world, and self-blame than victims of single-impact events, such as natural disasters or car accidents (Foa et al., 1999; Müller et al., 2010). Victims may also develop symptoms such as emotional dysregulation and difficulties in establishing interpersonal relationships. Therefore, alongside PTSD symptoms (reexperience, hypervigilance, avoidance), some individuals are prone to a set of potential symptoms of disorganization that are absent from the basic diagnosis. This pathological profile is called complex PTSD (Herman, 2015).

Recent analyses of PTSD symptoms and their characteristic effects on survivors of complex trauma have corroborated the notion of complex PTSD as an independent diagnosis (Cloitre et al., 2014; Redican et al., 2021). The International Classification of Diseases (ICD-11; WHO, 2018) classifies reactions to posttraumatic events as PTSD or complex PTSD (CPTSD). CPTSD includes symptoms of PTSD and disturbances of self-organization (DSO). DSO encompasses affective dysregulation, negative self-concept, and interpersonal difficulties. Both diagnoses require symptoms to cause significant impairment in relevant areas of social or occupational functioning.

The International Trauma Questionnaire (ITQ) was developed to measure symptoms of PTSD and CPTSD, as described by the ICD-11. To date, the ITQ is the only measure for CPTSD (Cloitre et al., 2018). The ITQ assesses six dimensions: reexperience (RE), hypervigilance (HV), and avoidance (AV) as PTSD symptoms; and affective dysregulation (AD), negative self-concept (NSC), and disturbances in relationships (DR) as DSO symptoms. Two-factor structures have shown better adjustment in the literature: a model with six correlated factors and a model containing two second-order dimensions corresponding to PTSD and DSO (Redican et al., 2021). However, a hierarchical model is underidentified for ITQ, as it estimates second-order factors from three first-order factors (Kline, 2023). In other words, both models (hierarchical and six hierarchical factors) are equivalent.

The PTCI assess three dimensions: cognitions about the self, the world, and self-blame. The original version has 33 items and was adapted to several cultures, demographics, and trauma types (Barzgar et al., 2023; Lebel et al., 2022). Nevertheless, its version exhibits an unstable factor structure, with further examination suggesting a reduction (Beck et al., 2004; Hyland et al., 2015) or an increase (Sexton et al., 2018) in items. Wells et al. (2019) developed a 9-item version of the PTCI (PTCI-9) to resolve instrument instability, excluding items showing semantic redundancy and residual correlations. The PTCI-9 shows superior adjustment indices for samples exposed to different traumatic events (Serier et al., 2023; Whitemann et al., 2022) and cultures (Barzgar et al., 2023; Zhan et al., 2024).

Similar to the ITQ, the proposed model for the PTCI-9 estimates a general factor from the three first-order dimensions (Wells et al., 2019), but it is also underidentified. In military and civilian samples, the three correlated factors of the PTCI-9 show adequate adjustment, superior to more extensive versions of the instrument, in addition to being invariant for biological sex (Serier et al., 2023; Whitemann et al., 2022). Women are more likely to be diagnosed with PTSD than men (Lehavot et al., 2018). Tolin and Foa’s (2002) cognitive model of PTSD proposed that differences in how men and women think about trauma after it has occurred (their trauma-related cognitions) may be a contributing factor. Trauma-exposed women endorsed more negative posttraumatic cognitions in a national sample of U.S. adults (Cox et al., 2014). Psychological constructs and the associated tests of these constructs may differ as a function of gender identity. Gender, as a psychological construct, impacts responses to trauma (Street & Dardis, 2018) and could influence how men and women describe posttraumatic cognitions. Measurement invariance examines whether a test measures similar constructs across groups and is an essential prerequisite for identifying group differences (Vandenberg & Lance, 2000).

The original model proposed for ITQ and tested in a United Kingdom sample (Cloitre et al., 2018) was replicated in different ethnic realities, such as in samples from the United States and Germany (Karatzias et al., 2017); France (Peraud et al., 2022); Nigeria, Kenya, and Ghana (Owczarek et al., 2020); Lebanon (Vallières et al., 2018); China, Hong Kong, Japan, and Taiwan (Ho et al., 2020); and Chile (Fresno et al., 2023). Despite the acceptable adequacy of the ITQ model, the low reliability of the AD items is a persistent issue in different ITQ versions, with the authors highlighting the necessity of expanding the number of items and developing a multidimensional model for the factor. In fact, the AD factor encompasses content related to hyperactivation and hypoactivation symptoms (Cyr et al., 2022; Sele et al., 2020; Vallières et al., 2018). The original ITQ model and the reliability of the AD factor remain to be investigated in a Portuguese-speaking sample.

The PTCI-9 has been validated in only three countries: Iran (Barzgar et al., 2023), China (Zhan et al., 2024), and the United States (Wells et al., 2019; Whiteman et al., 2022). The PTCI-9 presented a better fit than its extended version in all the samples. However, the Iranian version of the PTCI-9 showed inadequate adjustment, possibly due to translation issues. In Iran, item 3 did not load in the intended factor “Cognitions About Self-Blame”. Instead, it was related to “Cognitions About the Self”. The authors pointed out that the item 3 content (“Somebody else would not have gotten into this situation.”) does not reflect self-blame in the Persian language and that the translation should be revised in future studies (Barzgar et al., 2023). The PTCI-9 remains to be validated in a Latin American country.

The ITQ and PTCI-9 were translated into Brazilian Portuguese (Donat et al., 2019; Sbardelloto et al., 2013). However, the evidence of their reliability, validity related to external variables and internal structure, and measurement invariance across Brazilian samples still lacks investigation. Given the extent of the Brazilian territory and population, as well as the cultural diversity within the country, it is essential that a measure adequately assesses the construct in different regions. Additionally, people of different genders, ethnic backgrounds, age groups, and instruction levels should understand the items similarly, permitting group comparisons. Despite previous studies having included samples exposed to various trauma types (Wells et al., 2019; Whitemann et al., 2022) and presenting evidence for invariance for interpersonal and non-interpersonal traumas (Zhan et al., 2024), the PTCI-9 lacks evidence of invariance for various trauma types, an assumption necessary for comparison. The ITQ shows full invariance between clinical and nonclinical samples (Rácz et al., 2023), but its invariance for sociodemographic variables and traumatic events remains untested. Therefore, this study aimed to investigate the psychometric properties of the PTCI-9 and the ITQ. The hypotheses are as follows:

- Hypothesis 1: The six correlated factors model for ITQ will present adequate adjustment for all indices, congruent with previous investigations (Redican et al., 2021).
- Hypothesis 2: The three correlated factors for the PTCI-9 will show adequate adjustment for all indices, as in the original development study (Wells et al., 2019).
- Hypothesis 3: The ITQ and PTCI-9 will present full invariance for sociodemographic data in the Brazilian sample, as they have shown invariance in previous investigations of clinical and nonclinical samples (Rácz et al., 2023), gender and type of traumatic event (Serier et al., 2023; Zhan et al., 2024).
- Hypothesis 4: Posttraumatic cognitions and depressive symptoms will correlate with all six dimensions of the ITQ, with larger effect sizes for DSO symptoms (Camden et al., 2023; Herman, 2015; Serier et al., 2023).
- Hypothesis 5: Consistent with previous studies, women will show higher levels of PTSD and CPTSD symptoms (Cloitre et al., 2019).
- Hypothesis 6: Women will also display higher levels of posttraumatic cognitions (Cox et al., 2014; Serier et al., 2023).
- Hypothesis 7: People who experience trauma characterized by interpersonal violence will display higher levels of DSO symptoms and posttraumatic cognitions (Herman, 2015).

## Method

### Participants

An online questionnaire was administered via Google Forms (Google, LLC.), and 2,128 people responded to the survey. The inclusion criteria were having experienced a stressful event (with or without a self-reported diagnosis), being 18 years old or older, and being Brazilian. Participants who reported diagnoses that presented psychotic symptoms or did not describe a traumatic event were excluded from the analysis. Ten participants were not included in the analysis because they were not Brazilian, and 7 participants were excluded because they did not describe a traumatic event or reported not having experienced trauma in their lifetime. Therefore, data from 2,111 people (67.74% women) were included for analysis. Regarding gender, 0.86% of the sample declared identification with gender identities other than male and female (agender = 0.05%, nonbinary = 0.57%, gender neutral = 0.05%, none = 0.05%, and queer = 0.09%). Regarding race, 0.71% of the sample declared identification with other races (indigenous = 0.33%, yellow = 0.05%, caboclo = 0.05%, Latinos = 0.09%, and mestizo = 0.05%). A total of 0.05% of participants preferred not to declare their race or gender identities. The mean age was 28.60 years (SD = 9.25). For invariance analyses, participants were divided into three age groups: 18 to 24 years old (*n* = 926), 25 to 35 years old (*n* = 782), and 36 to 55 years old (*n* = 410). Table 1 summarizes the sociodemographic data of the sample, and Table 2 shows the frequency of reported trauma types.

**Table 1:** Sociodemographic data

Category	n	%	m (sd)								
			re	hv	av	ad	nsc	dr	self	world	sb
Gender											
Feminine	1432	67.84%	1.55 (1.28)	1.83 (1.37)	1.95 (1.27)	1.88 (1.14)	1.59 (1.41)	1.94 (1.31)	2.50 (1.50)	4.43 (1.57)	3.09 (1.70)
Masculine	650	30.79%	1.20 (1.26)	1.44 (1.29)	1.59 (1.35)	1.69 (1.16)	1.46 (1.36)	1.83 (1.33)	2.41 (1.53)	4.17 (1.60)	3.32 (1.81)
Other genres	28	1.33%	1.68 (1.33)	2.16 (1.31)	2.48 (1.30)	2.45 (1.24)	2.34 (1.27)	2.82 (1.12)	3.26 (1.61)	5.75 (1.18)	3.85 (1.72)
NA	1	0.047%	-		-	-	-	-	-	-	-
Race											
White	1201	56.89%	1.42 (1.29)	1.65 (1.35)	1.77 (1.30)	1.76 (1.15)	1.52 (1.39)	1.89 (1.34)	2.43 (1.47)	4.29 (1.61)	3.21 (1.78)
Brown (*Parda*)	635	30.08%	1.43 (1.28)	1.73 (1.35)	1.92 (1.32)	1.89 (1.14)	1.61 (1.40)	1.93 (1.29)	2.52 (1.55)	4.52 (1.53)	3.18 (1.68)
Asian	14	0.66%	1.79 (1.01)	2.14 (1.18)	2.07 (1.09)	2.79 (1.07)	2.14 (1.31)	2.93 (0.94)	3.02 (1.36)	4.83 (1.38)	2.86 (1.60)
Black	228	10.80%	1.57 (1.31)	1.91 (1.37)	1.99 (1.31)	1.91 (1.17)	1.61 (1.42)	1.97 (1.30)	2.53 (1.59)	4.34 (1.57)	2.96 (1.65)
Indigenous	7	0.33%	1.64 (1.31)	1.93 (1.06)	2.36 (0.99)	2.36 (1.25)	1.43 (0.89)	2.07 (0.89)	3.29 (1.18)	4.86 (1.18)	3.29 (2.05)
Other races	7	0.76%	1.21 (1.08)	1.50 (1.61)	1.50 (1.41)	1.21 (1.25)	1.14 (1.65)	0.86 (1.44)	1.81 (1.10)	3.38 (1.01)	2.24 (2.19)
NA	1	0.05%	-	-	-	-	-	-	-	-	-
Instruction level											
Basic degree (complete and incomplete)	110	5.21%	1.73 (1.30)	1.89 (1.30)	2.09 (1.42)	1.94 (1.06)	1.80 (1.41)	2.08 (1.29)	2.76 (1.46)	4.59 (1.52)	3.17 (1.71)
Undergraduate degree (incomplete)	1060	50.21%	1.53 (1.32)	1.81 (1.37)	1.96 (1.29)	1.97 (1.16)	1.71 (1.40)	2.08 (1.34)	2.64 (1.56)	4.51 (1.54)	3.23 (1.73)
Undergraduate degree (complete)	218	10.33%	1.40 (1.30)	1.80 (1.34)	1.93 (1.32)	1.92 (1.20)	1.70 (1.45)	2.08 (1.35)	2.57 (1.61)	4.47 (1.61)	3.44 (1.81)
Graduate degree (incomplete and complete)	730	34.58%	1.29 (1.21)	1.51 (1.33)	1.62 (1.28)	1.57 (1.10)	1.27 (1.32)	1.62 (1.24)	2.18 (1.37)	4.11 (1.61)	3.01 (1.72)
Brazilian region											
Southeast	932	44.15%	1.41 (1.27)	1.68 (1.34)	1.79 (1.30)	1.80 (1.14)	1.60 (1.42)	1.88 (1.29)	2.43 (1.48)	4.35 (1.56)	3.26 (1.77)
South	342	16.20%	1.50 (1.29)	1.68 (1.40)	1.84 (1.29)	1.78 (1.15)	1.52 (1.32)	1.97 (1.37)	2.44 (1.41)	4.31 (1.65)	3.14 (1.74)
Midwest	195	9.24%	1.61 (1.27)	1.77 (1.33)	1.94 (1.30)	1.90 (1.23)	1.52 (1.41)	1.99 (1.32)	2.44 (1.45)	4.30 (1.64)	3.13 (1.77)
North east	501	23.73%	1.44 (1.33)	1.80 (1.37)	1.94 (1.32)	1.89 (1.16)	1.58 (1.40)	1.97 (1.36)	2.62 (1.63)	4.47 (1.55)	3.12 (1.68)
North	141	6.68%	1.35 (1.23)	1.63 (1.35)	1.76 (1.33)	1.80 (1.17)	1.37 (1.36)	1.77 (1.30)	2.48 (1.65)	4.45 (1.59)	2.97 (1.71)

*%* Percentage of the sample in a given category, *ad* Affective dysregulation, *av* Avoidance, *dr* Disturbance in relationships, *hv* Hypervigilance, *m* Mean, *n* Number of participants, *na* Not available, *nsc* Negative self-concept, *re* Reexperience, *sb* Cognitions About Self-Blame, *sd* Standard deviation, *self* Cognitions About the Self, *world* Cognitions About the World

**Table 2:** Frequency of self-reported trauma categories

Trauma category	n	%	m (sd)								
			re	hv	av	ad	nsc	dr	self	world	sb
Death of a close one	298	14.12%	1.44 (1.24)	1.59 (1.25)	1.47 (1.32)	1.71 (1.16)	1.43 (1.37)	1.84 (1.34)	2.47 (1.54)	4.12 (1.67)	2.38 (1.53)
Domestic violence and family conflicts	235	11.13%	1.45 (1.28)	1.89 (1.32)	1.43 (1.23)	1.76 (1.14)	1.49 (1.41)	2.04 (1.29)	2.53 (1.57)	4.31 (1.61)	3.72 (1.57)
Moral and sexual abuse	214	10.14%	1.79 (1.35)	2.23 (1.32)	2.34 (1.29)	2.21 (1.14)	1.90 (1.44)	2.27 (1.30)	2.69 (1.63)	4.90 (1.39)	3.29 (1.68)
Affective relationship related conflicts	210	9.95%	1.54 (1.31)	1.70 (1.33)	1.82 (1.44)	1.68 (1.13)	1.28 (1.35)	1.66 (1.27)	2.30 (1.36)	3.99 (1.63)	3.20 (1.79)
Exposure and humiliation	197	9.33%	1.35 (1.23)	1.87 (1.33)	1.66 (1.29)	1.95 (1.19)	1.68 (1.34)	2.12 (1.32)	2.50 (1.56)	4.61 (1.50)	3.66 (1.70)
Problems of close ones	190	9.00%	1.42 (1.27)	1.76 (1.32)	1.71 (1.40)	1.78 (1.08)	1.44 (1.34)	1.78 (1.28)	2.29 (1.31)	4.28 (1.56)	2.34 (1.51)
Stressful routine	186	8.81%	1.33 (1.30)	1.88 (1.30)	1.61 (1.33)	1.64 (1.13)	1.48 (1.38)	1.69 (1.34)	2.49 (1.56)	4.49 (1.60)	3.48 (1.81)
Physical and mental health issues	177	8.38%	1.29 (1.31)	1.71 (1.30)	1.63 (1.31)	1.80 (1.23)	1.61 (1.42)	1.88 (1.37)	2.64 (1.55)	4.20 (1.64)	3.35 (1.84)
Academic related stresses	173	8.20%	1.44 (1.31)	1.75 (1.26)	1.49 (1.30)	1.80 (1.14)	1.62 (1.38)	1.76 (1.26)	2.38 (1.45)	4.37 (1.40)	3.82 (1.69)
Work and finances	157	7.44%	1.48 (1.28)	2.18 (1.27)	2.09 (1.39)	2.01 (1.14)	1.80 (1.43)	2.13 (1.35)	2.61 (1.54)	4.65 (1.52)	3.04 (1.62)
Others	74	3.51%	1.12 (1.04)	1.60 (1.16)	1.24 (1.18)	1.47 (0.99)	1.14 (1.37)	1.57 (1.19)	2.10 (1.40)	3.87 (1.64)	3.16 (1.84)

*%* Percentage of the sample in a trauma category, *n* Number of participants

### Instruments

#### Sociodemographic questionnaire

This questionnaire contains questions about participants’ age, gender, race, income, occupation, and instruction level.

#### Adverse situation descriptive questionnaire

This questionnaire was developed for the Brazilian population by Campos and Trentini (2019). It includes an open-ended item inviting respondents to describe the most stressful situation they faced during their lifetime. Participants are asked to report the time that has passed since the event, the stress level felt during the event and in the present, and the social support received after the event.

#### ITQ

The ITQ is the gold standard for assessing symptoms of PTSD and complex PTSD. The ITQ comprises ten items assessing six domains: HV, AV, RE, AD, DR, and NSC. Participants respond to items (symptoms) about how much they have been disturbed by them in the last few months on a 5-point ordered response scale: 0 (“not at all”) - 4 (“extremely”; Cloitre et al., 2014). The ITQ was adapted for Brazil by Donat et al. (2019), but the psychometric properties of the Brazilian ITQ have not yet been investigated.

#### PTCI-9

The PTCI-9 investigates cognitions about the self, the world, and self-blame. Respondents rate their level of agreement with the items on a seven-point ordered response type scale: 1 (“completely disagree”) - 7 (“completely agree”; Foa et al., 1999). The original items were translated into the Brazilian Portuguese (Sbardelloto et al., 2013). However, the external and internal validity of the Brazilian PTCI-9 have not been investigated.

#### Patient Health Questionnaire 9 (PHQ-9)

The PHQ-9 includes nine questions that assess symptoms of an episode of major depression according to the DSM-5. Symptoms consisted of depressed mood, anhedonia, problems with sleep, tiredness or lack of energy, changes in appetite or weight, feelings of guilt or worthlessness, problems concentrating, feelings of sluggishness or restlessness, and suicidal thoughts. Respondents evaluate the items based on their frequency in the past 2 weeks on a four-point scale (0 - “none of the time” to 3 “almost every day”; Kroenke et al., 2001). The PHQ-9 was adapted for Brazil by Santos et al. (2013). In our sample, the PHQ-9 presented adequate reliability (ω = 0.85).

### Procedure

Before data collection, we obtained permission to use and validate the ITQ and PTCI-9 from the respective original authors. We performed a confirmatory factor analysis (CFA) to evaluate the plausibility of correlated factors for the Brazilian versions of the ITQ and PTCI-9 instruments. We implemented the robust diagonal weighted least squares (RDWLS) estimation method, which is suitable for categorical data (Li, 2016). The following adjustment indices were used: χ2; χ2/df; comparative fit index (CFI), Tucker‒Lewis index (TLI), standardized root mean residual (SRMR), and root mean square error of approximation (RMSEA). A good fit is indicated by the following parameters: nonsignificant χ2 values; the χ2/df ratio must be < 5 or, preferably, < 3; CFI and TLI values must be > 0.90 and preferably > 0.95; RMSEA values should be < 0.08 or, preferably < 0.06, with a confidence interval (upper limit) < 0.10 (Hu & Bentler, 1999). We assessed the reliability of the ITQ and PTCI-9 using McDonald’s Ômega (ω). We considered a ω > 0.70 to indicate adequate reliability (Beland et al., 2017).

We carried out multigroup CFAs (AFCMG) to investigate the invariance of the ITQ and PTCI-9 for the following variables: gender, age group, ethnicity, per capita income, location (Brazilian region), and trauma type. We implemented the RDWLS estimation method (Li, 2016). The AFCMG evaluates the measurement invariance in three models: configural, metric, and scalar. Measurement invariance involves an iterative process in which additional constraints are imposed on the model to determine whether model parameters are equivalent across groups (Vandenberg & Lance, 2000). Configural invariance tests the equivalence of factor structures. Metric invariance tests the equivalence of factor loadings (i.e., do indicators relate to the latent construct similarly?) across groups. Scalar invariance tests the equivalence of item intercepts (i.e., do mean differences on the latent factor reflect mean differences on the items?). A lack of scalar invariance suggests that the items may function differently across groups and that these differences are unrelated to latent factor differences. We assessed measurement invariance using the CFI difference test (ΔCFI). When setting a parameter, if a significant reduction in CFI indices is found (ΔCFI > 0.01), measurement invariance cannot be accepted (Cheung & Rensvold, 2002).

The relationships between the ITQ and PTCI-9 dimensions with each other and with depression were tested using Pearson correlations, with Bonferroni correction for multiple comparisons. We performed Student’s t test for independent samples to investigate the extent to which posttraumatic cognition, PTSD, and CPTSD levels differed between men and women. One-way analysis of variance (ANOVA) was carried out to evaluate whether there were differences in the levels of CPTSD among the different trauma types. The assumption of homogeneity of variance was assessed using Levene’s test (Field, 2013). We performed all analyses in R (RStudio Team, 2020).

### Ethical considerations

The present study is part of a larger project approved by the Research Ethics Committee of the Center for Philosophy and Human Sciences at the Federal University of Rio de Janeiro (CAAE nº 49455821.1.0000.5582). The data and documents from this study were stored for at least 5 years. This research was performed in accordance with the guidelines and standards of resolutions 466/2012 and 510/2016 of the National Health Council (2012, 2016). All participants signed a free and informed consent form with detailed information on the technical and ethical aspects of the research.

## Results

The three-factor correlated model for the PTCI-9 presented excellent fit indices, except for the RMSEA, with χ2(24) = 769.603 (*p* < 0.0001), CFI = 0.954, TLI = 0.931, RMSEA = 0.121 (CI = 0.114-0.129), and SRMR = 0.068. An inspection of the highest modification indices (MI) indicated a residual correlation between items 1 and 7 (MI = 25.743), relating to self-responsibility, and between item 3 and cognitions about the world (MI = 23,648). A possible explanation lies in the semantics of item 3 (“Another person would have prevented the event from occurring”), which is similar to other Cognitions About the World, which also include the term “person/people”. The three dimensions of the PTCI-9 presented ω greater than 0.70 (cognitions about the self = 0.8, about the world = 0.81, about self-responsibility = 0.75). For the ITQ, the model of six correlated factors presented adequate adjustment to the data, with χ2(39) = 473.741 (*p* < 0.0001), CFI = 0.986, TLI = 0.978, RMSEA = 0.073 (CI = 0.067-0.078), and SRMR = 0.031. Table 3 presents the tested models and their respective factor loadings. Regarding internal consistency, except for AD, all ω CR indices were above the suggested cutoff point of 0.70, with RE = 0.79, AV = 0.80, HV = 0.85, AD = 0.62, NSC = 0.92, and DR = 0.86.

**Table 3:** Factor loadings and descriptive statistics for PTCI-9 e ITQ items

Items	Standardized factor loadings						m	sd
	ptci-9							
	self		w		sb			
01					0.68		2.90	2.13
03					0.65		3.51	2.20
07					0.80		3.11	2.16
02			0.77				4.44	1.87
04			0.77				3.56	2.01
06			0.68				5.11	.174
05	0.73						2.24	1.73
08	0.99						3.18	2.12
09	0.77						2.01	1.58
	itq							
	re	av	hv	ad	dr	nsc		
01	0.77						1.24	1.42
02	0.85						1.65	1.49
03		0.80					1.80	1.42
04		0.80					1.89	1.50
05			0.86				1.90	1.52
06			0.89				1.52	1.45
07				0.65			2.02	1.34
08				0.67			1.62	1.42
09					0.90		1.61	1.49
10					0.93		1.51	1.45
11						0.93	2.01	1.43
12						0.79	1.82	1.45

*ad* Affective dysregulation, *av* Avoidance, *dr* Disturbance in relationships, *hv* Hypervigilance, *itq* International Trauma Questionnaire, *m* Mean, *n* Number of participants, *na* Not available, *nsc* Negative self-concept, *ptci-9* Post-Traumatic Cognitions Inventory-9, *re* Reexperience, *sb* Cognitions About Self-Blame, *sd* Standard deviation, *self* Cognitions About the Self, *world* Cognitions About the World

For AFCMG analysis, only men and women were considered (for gender), and only white, brown, and Black individuals were considered (for race). Patients of other sexes and races were not included due to the small sample size. Both instruments demonstrated scalar invariance for all sociodemographic variables investigated (gender, age, race, region, and income), and the ITQ also showed scalar invariance for trauma type. PTCI-9, however, presented only metric invariance for trauma type. An inspection of MIs revealed that the highest MIs were between item 3 of the PTCI-9 and the latent factor cognitions about the world and cognitions about the self for all groups, with MIs ranging between 10.280 and 59.250. This suggests that victims of those types of trauma possibly understand the items differently, and group comparisons using the Brazilian PTCI-9 for trauma types might not be recommended. The results of the AFCMGs for the PTCI-9 and ITQ are shown in Table 4.

**Table 4:** Multigroup confirmatory factor analysis for PTCI-9 and ITQ

	Goodness-of-fit indexes									
	ptci-9					itq				
	rmsea (90% ic)	srmr	tli	cfi	Δcfi	rmsea (90% ic)	srmr	tli	cfi	Δcfi
Sex										
Configural	0.118 (0.111–0.126)	0.067	0.933	0.955	-	0.071 (0.065–0.077)	0.032	0.977	0.986	-
Metric	0.106 (0.099–0.114)	0.068	0.946	0.959	+0.004	0.062 (0.056–0.068)	0.033	0.983	0.989	+0.003
Scalar	0.088 (0.082–0.093)	0.068	0.963	0.951	0.008	0.061 (0.056–0.066)	0.032	0.983	0.985	0.004
Age groups										
Configural	0.118 (0.110–0.125)	0.069	0.934	0.956	-	0.074 (0.068–0.080)	0.034	0.974	0.985	-
Metric	0.104 (0.097–0.111)	0.070	0.949	0.960	+0.004	0.066 (0.060–0.072)	0.038	0.979	0.986	+0.011
Scalar	0.081 (0.076–0.086)	0.068	0.969	0.951	0.009	0.056 (0.051–0.061)	0.035	0.985	0.985	0.000
Trauma type										
Configural	0.121 (0.113–0.129)	0.075	0.933	0.955	-	0.073 (0.066–0.080)	0.043	0.977	0.986	-
Metric	0.104 (0.097–0.112)	0.082	0.950	0.959	+0.004	0.061 (0.054–0.068)	0.049	0.984	0.989	+0.003
Scalar	0.078 (0.073–0.084)	0.078	0.972	0.947	0.012	0.056 (0.050–0.062)	0.044	0.986	0.985	0.004
Instruction level										
Configural	0.120 (0.113–0.128)	0.069	0.931	0.954	-	0.075 (0.069–0.081)	0.033	0.974	0.985	-
Metric	0.105 (0.101–0.115)	0.070	0.945	0.959	+0.005	0.066 (0.060–0.072)	0.035	0.980	0.987	+0.002
Scalar	0.085 (0.080–0.091)	0.070	0.965	0.954	0.005	0.058 (0.053–0.063)	0.033	0.974	0.987	0.000
Race										
Configural	0.123 (0.116-.131)	.070	.929	.952	-	0.069 (0.062–0.075)	0.033	0.979	0.988	-
Metric	0.105 (0.098-.113)	.071	.948	.959	+.007	0.058 (0.052–0.064)	0.035	0.985	0.990	+0.002
Scalar	0.074 (0.069-.088)	.071	.974	.960	+.001	0.053 (0.048–0.058)	0.033	0.988	0.988	0.002
Region										
Configural	.125 (0.117–0.135)	0.072	0.930	0.954	-	0.071 (0.065–0.077)	0.035	0.978	0.987	-
Metric	.106 (0.099–0.113)	0.075	0.950	0.960	+0.006	0.057 (0.050–0.063)	0.037	0.986	0.991	+0.004
Scalar	.073 (0.068–0.078)	0.074	0.976	0.958	0.002	0.049 (0.044–0.055)	0.035	0.989	0.989	0.002

*Δcfi* Difference in CFI for the previous model, *cfi* Comparative Fit Index, *itq* International Trauma Questionnaire, *ptci-9* Post-Traumatic Cognitions Inventory-9, *rmsea* Mean Square Error of Approximation, *srmr* Standardized Root Mean Square Residual, *tli* Tucker-Lewis Index

As predicted, all three posttraumatic cognition dimensions were correlated with PTSD, DSO, and depression, with stronger correlations with DSO domains. Cognitions about self-blame had a more significant effect size for DR (*r* = 0.61, *p* < 0.001), cognitions about self had a more significant effect size for NSC (*r* = 0.68, *p* < 0.001), and cognitions about the world had a more significant effect size for DR (*r* = 0.49, *p* < 0.001). Table 5 displays the correlation matrix for all variables.

**Table 5:** Validity related to relationship with external variables

	1	2	3	4	5	6	7	8	9	10
1. Cognitions about the world	-									
2. Cognitions about self-blame	0.26*	-								
3. Cognitions about the self	0.37*	0.49*	-							
4. Reexperience	0.18*	0.31*	0.40*	-						
5. Avoidance	0.26*	0.38*	0.39*	0.54*	-					
6. Hypervigilance	0.15*	0.35*	0.38*	0.57*	0.56*	-				
7. Affective dysregulation	0.23*	0.46*	0.54*	0.45*	0.44*	0.46*	-			
8. Negative self-concept	0.33*	0.44*	0.68*	0.42*	0.41*	0.42*	0.62*	-		
9. Disturbances in relationships	0.24*	0.49*	0.52*	0.36*	0.40*	0.36*	0.69*	0.58*	-	
10. Depression	0.23*	0.44*	0.59*	0.46*	0.43*	0.47*	0.61*	0.62*	0.51*	-

^*^P < .001

Women showed higher values of RE (t(2080) = 5.82; *p* < 0.001; d = 0.28), AV (t(2080) = 5.88; *p* < 0.001; d = 0.28), HV (t(2080) = 6.062; *p* < 0.001; d = 0.29), AD (t(2080) = 3.69; *p* < 0.001; d = 0.16) and NSC (t(2080) = 2.06; *p* < 0.039; d = 0.10). Regarding posttraumatic cognitions, women also showed higher levels of cognitions about the world (t(2080) = 3.51; *p* < 0.001; d = 0.17), and men showed a higher level of cognitions about self-blame (t(2080) = -2.80; *p* < 0.01; d = -0.13). DR (t(2080) = 1.77; *p* > 0.05) and cognition about the self did not significantly differ by gender (t(2080) = 1.23; *p* > 0.05). For the relationship between trauma type and DSO domains, Levene’s tests indicated homogeneity of variance for AD (F(2100) = 1.42, *p* > 0.05), for NSC (F(2100) = 0.88, *p* > 0.05) and for DR (F(2100) = 0.96, *p* > 0.05). ANOVA revealed significant differences between trauma types for AD (F(2100) = 5.16; *p* < 0.001; N2 = 0.024). Post hoc tests indicated small but significant differences in abuse/harassment with academic-related stresses (t(2100) = 3.55; *p* < 0.05; d = 0.36), diseases/hospitalization (t(2100) = 3.53; *p* < 0.05; d = 0.36), death and grief (t(2100) = 4.89; *p* < 0.001; d = 0.44), family conflicts (t(2100) = 3.78; *p* < 0.05; d = 0.38), relationship-related conflicts (t(2100) = 4.08; *p* < 0.05; d = 0.40), routine-related conflicts (t(2100) = 4.65; *p* < 0.001; d = 0.47), and work and finances-related conflicts (t(2100) = 4.80; *p* < 0.001; d = 0.50). For NSC (F(2100) = 4.19; *p* < 0.001; N2 = 0.02), small differences were found between abuse/harassment and death and grief (t(2100) = 3.80; *p* < 0.05; d = 0.34) and routine-related (t(2100) = 4.52; *p* < 0.001; d = 0.45) and between routine-related and domestic violence as victims or testimony/negligence (t(2100) = -2.23; *p* < 0.01; d = -0.37). For DR (F(2100) = 5.11; *p* < 0.001; N2 = 0.024), there were moderate differences between abuse/harassment and academic-related stress (t(2100) = 3.80; *p* < 0.05; d = 0.39), death and grief (t(2100) = 3.68; *p* < 0.05; d = 0.33), family conflicts (t(2100) = 3.71; *p* < 0.001; d = 0.37), routine-related stress (t(2100) = 4.63; *p* < 0.001; d = 0.46) and work and finance-related stress (t(2100) = 4.22; *p* < 0.001; d = 0.44). There were also differences between humiliating experiences and routine-related stress (t(2100) = 3.46; *p* < 0.05; d = 0.35) and between domestic violence as a victim or testimony/negligence and routine-related stress (t(2100) = 3.65; *p* < 0.05; d = 0.36). All post hoc analyses for the trauma type results are displayed in the Supplementary Material.

## Discussion

The main objectives of the study were to investigate the psychometric properties of the Brazilian versions of two measures of trauma-related dimensions in psychopathology, namely, the PTCI-9 and the ITQ. Both measures are relevant for assessing CPTSD symptoms, as they are the only measures available in Brazilian Portuguese. The present study advances the current knowledge by investigating internal structure validity and relationships with external variables, reliability, and measurement invariance for sociodemographic variables (gender, age groups, race, education level, and Brazilian region) and trauma types in a Brazilian sample. The favorable psychometric properties and validity evidence presented here suggest the use of the PTCI-9 and ITQ in Brazil.

Regarding hypothesis 1, the original ITQ model was confirmed (Cloitre et al., 2018). The six correlated factors model showed excellent adjustment to the data. Therefore, the validity of the internal structure of the Brazilian ITQ is corroborated. Replications in different cultures are warranted because they may expand the evidence for reasonable adjustment (Redican et al., 2021). All factors except for AD showed adequate reliability indices. The lower reliability of the AD factor (ω = 0.62) probably indicates limitations to its unidimensionality. Affective dysregulation, or emotion dysregulation, is a multidimensional construct that comprises different maladaptive strategies for regulating emotions (Sloan et al., 2017). Further development of the ITQ should address this issue by developing a multidimensional affective dysregulation factor, with items regarding different failed strategies for regulating one’s emotions.

For hypothesis 2, the three correlated factors PTCI-9 showed adequate adjustment for all indices (except for RMSEA). Other studies also corroborated this model, proving that it is superior to the original version of the PTCI (Wells et al., 2019). Our results add to the validity evidence internationally, based on a different cultural reality. However, in contrast to hypothesis 2, our study indicated a high RMSEA value for the instrument, above the cutoff point of 0.080 (Hu & Bentler, 1999). This result contradicts the findings of studies in which the scale was originally written. In samples with diverse trauma types (Whitemann et al., 2022) and from veteran men and civilian women (Serier et al., 2023), the RMSEA values ranged between 0.050 and 0.062. In addition to the Brazilian version, the Persian PTCI-9 showed a slightly greater RMSEA (0.085) for the general population (Barzgar et al., 2023). The high RMSEA for the PTCI-9 may reflect a semantic redundancy of item 3 (“another person would have prevented the event from happening”), and the cognitions about the world factor. The factor cognitions about the world also includes items related to the word person/people. As versions in the original language did not show high RMSEAs, this is likely a translation issue. Item 3, in the original language, does not include the word “people” (“Somebody else would not have gotten into this situation”). A possible solution to this problem is the reformulation of the translated item.

Regarding hypothesis 3, the scalar invariance for sex, age, race, Brazilian region, and instruction level was corroborated for the PTCI-9 and ITQ. The ITQ also showed full invariance for trauma type. Our study thus expands on previous findings that investigated invariance only between clinical and nonclinical samples (Nielsen et al., 2023; Rácz et al., 2023). The PTCI-9 showed only metric invariance for trauma type, not corroborating hypothesis 3. Investigating the highest MIs in the scalar model revealed that item 3 presented unexpected correlations with the other two factors. The same item also showed a high MI for the configural model. We hypothesize that after the reformulation of item 3, adequate RMSEA and invariance for trauma type will be attained. Another possible explanation lies in the potential differential impact of posttraumatic cognitions on different traumatic events. Cognitions about the self, the world, and self-blame are particularly pervasive for complex traumas involving interpersonal aggression and internalization of shame and guilt, such as sexual abuse, exposure to humiliating situations, and domestic violence (Herman, 2015). Another potential cause of not achieving full invariance for trauma type is the small sample size for each trauma group, which is less than 200 for most groups (Kyriazos, 2018).

Our study also identified more substantial effect sizes for the relationship between posttraumatic cognitions and DSO than for the relationship between posttraumatic cognitions and PTSD, corroborating hypothesis 4. Regarding Hypotheses 5 and 6, all variables showed differences for gender, with higher levels for women, except for DR and cognitions about the self. Men had higher levels of cognitions about self-blame, contrary to findings in a national U.S. sample (Cox et al., 2014). Congruent with hypothesis 7, situations of abuse, a trauma that involves interpersonal violence and can be understood as complex, showed higher levels for all DSO domains than single-impact traumas (such as hospitalization and work and finances). Abuse is the major predictor of PTSD in women (Pico-Alfonso, 2005) and is a traumatic event associated with CPTSD symptoms (Zlotnick et al., 1996). Humiliating experiences also presented a greater level of DR than did routine-related events. It is hypothesized that humiliation affects victims’ beliefs and trust in the world (Leask, 2013).

One limitation of the present study is the sampling method. Our sample is heterogeneous and was obtained via an online survey, with most participants having a higher education level, a numerical minority in the Brazilian context, and less than 30% of the population having completed or incomplete education. In that sense, it is necessary to investigate evidence of the validity of instruments in specific samples to obtain indicators of measurement invariance for educational level. Furthermore, it is necessary to investigate the concurrent validity of the Brazilian ITQ with other PTSD measures and its predictive validity in clinical samples for both instruments in Brazilian samples. We recommend reforming the translated version of item 3 of the PTCI-9, in which the word “person” is substituted. For ITQ, the AD factor must be expanded to encompass a multidimensional model of emotional regulation. Finally, future studies should investigate both scales’ ability to correctly identify clinical and nonclinical Brazilian samples and clinical changes throughout treatment.

## Conclusion

This study presents evidence of the validity, reliability, and measurement invariance of the Brazilian versions of two trauma-related measures, the ITQ and PTCI-9. Beyond classical PTSD indicators (reexperience, avoidance, and hypervigilance), the ITQ addresses DSO, a cluster of symptoms present in CPTSD. The ITQ will enable symptoms of DSO and CPTSD, as conceptualized by the ICD-11, to be measured in Brazilian culture. Thus, this measurement instrument will expand the CPTSD literature in Brazil. The PTCI-9 measures posttraumatic cognitions about the self, the world, and self-blame. Trauma-related cognitions impact the development and maintenance of PTSD and CPTSD. The PTCI-9 can be used effectively in studies on PTSD and CPTSD in Brazilian individuals.

Generalizability is an important characteristic when evaluating any measurement instrument. This study contributes to the knowledge of the factor structure of the ITQ and PTCI-9 in different populations by presenting evidence of measurement invariance for gender, race, age groups, instruction levels, and the five Brazilian regions. The results show the importance of establishing the fundamental measurement properties before inferring the cross-cultural universality of the construct to be measured. In sum, the findings provide evidence to test the external validity and cross-cultural applicability of the conceptualization and operationalization of the PTSD, CPTSD, and posttraumatic cognitions constructs.

### Supplementary Information

**Supplementary Material 1.**

## Data Availability

The data that support the findings of this study are available on request from the corresponding author. The data are not publicly available due to privacy or ethical restrictions.

## Abbreviations

AD: Affective dysregulation
AFCMG: Multigroup confirmatory factor analysis
ANOVA: Analysis of variance
AV: Avoidance
CFA: Confirmatory factor analysis
CFI: Comparative fit index
CI: Confidence interval
CPTSD: Complex posttraumatic stress disorder
CR: Composite reliability
d: Cohen’s d
df: Degrees of freedom
DR: Disturbance in relationships
HV: Hypervigilance
ITQ: International trauma questionnaire
NSC: Negative self-concept
PTCI-9: Posttraumatic cognitions inventory-9
PTSD: Posttraumatic stress disorder
r: Correlation coefficient
RE: Reexperience
RMSEA: Root mean square error of approximation
sb: Cognitions About Self-Blame
self: Cognitions About the Self
SRMR: Root mean square residual and standardized root mean square residual
TLI: Tucker Lewis Index
world: Cognitions About the World
X2: Chi-squared test
η2: Eta-Squared
